# The prevalence of diagnosed specific back pain in primary health care in Region Västra Götaland: a register study of 1.7 million inhabitants

**DOI:** 10.1017/S1463423621000426

**Published:** 2021-08-11

**Authors:** Madeleine Kruse, Olof Thoreson

**Affiliations:** 1 Research and Development Primary Health Care Centre Gothenburg and Södra Bohuslän, Gothenburg, Sweden; 2 Wästerläkarna AB, Gothenburg, Sweden; 3 Department of Orthopedics, Institute of Clinical Sciences at Sahlgrenska Academy, University of Gothenburg, Gothenburg, Sweden

**Keywords:** disc herniation, primary health care, register, specific back pain, spinal stenosis

## Abstract

**Objectives:**

To evaluate the one-year prevalence of diagnosed specific back pain in Region Västra Götaland, inhabiting 1.7 million people.

**Designs:**

A retrospective register study.

**Settings:**

Data from 2014 to 2019 were extracted from the VEGA register, which holds all health data from all publicly funded health care establishments in Region Västra Götaland. Aggregated data are presented as the one-year prevalence of unique individuals diagnosed with International Statistical Classification of Diseases and Related Health Problems – Tenth Revision codes representing specific back pain.

**Subjects:**

All inhabitants in Region Västra Götaland.

**Main outcome measures:**

The one-year prevalence of diagnosed specific back pain stratified by age, sex, and health care level.

**Results:**

In 2019, the one-year prevalence of diagnosed specific back pain in public primary health care centres was 0.82%, rehabilitation care 0.35%, and the combined increase was 156% from 2014. In specialized health care, the diagnosed prevalence during 2014–2019 has remained relatively unchanged. The prevalence was significantly higher among women in primary health care and rehabilitation care. M48.0 (spinal stenosis) and M51.1K (lumbar disc herniation with radiculopathy) were the most common sub-classifications. For M48.0, prevalence increased by age, whereas M51.1K peaked within the 45–64 years category.

**Conclusions:**

The one-year prevalence of diagnosed specific back pain in primary health care was 1.17% in 2019 and has increased since 2014. Women were diagnosed considerably more frequently than men, which is not reflected in surgical treatment prevalence.

## Key points

- In 2019, the one-year prevalence of diagnosed specific back pain in primary health care was 1.17% (PHC 0.82% and rehabilitation care 0.35%) with an increase of 156% from 2014.
- Diagnosed specific back pain is more common among women than men in all health care levels, but significantly more dominant in primary health care.
- The study displays remarkable differences in sex prevalence between diagnosed specific back pain in primary health care and surgery interventions.

## Introduction

Back pain is a major problem throughout the world. It causes more disability than any other condition and is the leading cause of activity limitation and work absence globally (Hoy *et al.*, 2014). A systematic review showed a one-month global prevalence of activity-limiting low back pain of 23% (Hoy *et al.*, 2012). But are those suffering from the condition, even though it is so common, a forgotten and overlooked group?

Back pain can be divided into different categories: specific spinal pathology, nerve root pain/radicular pain, and nonspecific back pain. Nonspecific back pain, i.e., back pain that is not attributable to a recognizable, known specific pathology, is the most common (Airaksinen *et al.*, 2006, Koes, Van Tulder & Thomas, 2006). Around 10% of patients with back pain have specific spinal pathology or nerve root pain/radicular pain, i.e. specific back pain (Koes, Van Tulder & Thomas, 2006). There are many causes of specific back pain where spinal stenosis and disc herniation are the most treated pathological conditions (Schroeder, Kurd & Vaccaro, 2016; Hartvigsen *et al.*, 2018; Fritzell *et al.*, 2020). Specific back pain will therefore in this study refer to spinal stenosis and disc herniation at cervical/thoracic/lumbar levels with International Statistical Classification of Diseases and Related Health Problems – Tenth Revision (ICD-10) codes demonstrated in Appendix 1 (Organization, 1992).

Spinal stenosis is characterized by a narrowing of the space for the spinal canal at the cervical, thoracic, or lumbar level, leading to a compression of the spinal cord or nerve roots. Spinal stenosis is mainly caused by degenerative changes and is thereby more prevalent with increased age (Melancia, Francisco & Antunes, 2014; Schroeder, Kurd & Vaccaro, 2016). Aside from age, being overweight is another risk factor (Knutsson *et al.*, 2015). Studies imply a large range in the reported prevalence of lumbar spinal stenosis: in primary health care, the prevalence in a European, North American, and Japanese population was 15% based on radiological diagnosis and 25% on clinical diagnosis (Schroeder, Kurd & Vaccaro, 2016; Jensen *et al.*, 2020).

Disc herniation (cervical, thoracic, and lumbar) occurs when the nucleus pulposus protrudes through the annulus fibrosus. This nerve compression may result in pain and other neurological symptoms, such as weakness or numbness. Proposed risk factors are debated but include male sex, cigarette smoking, and heavy lifting (Wong *et al.*, 2014). Lumbar disc herniation is the most common location and affects 1% to 5% of the population annually (Jordan, Konstantinou & O’Dowd, 2011; Arts *et al.*, 2019).

The healthcare system in Sweden is divided into primary healthcare, specialized outpatient care, and inpatient healthcare. All inhabitants in Sweden have equal access to healthcare services according to the Health and Medical Service Act. Health care providers are mostly public, but public-funded private establishments have expanded considerably over the past few years and all act under the same economical rules and structures. The primary health care, consisting of primary health care centres (PHC) and rehabilitation centres, is usually the first health care provider that patients visit and where the primary diagnosis is made (Riksdag, 2017). Patients can also attend the hospital emergency departments and outpatient clinics with or without a referral. The ICD-10 system is used to code all health care visits and diagnoses. Public health care is mainly funded through general taxation, whereas payments for patient visits and performance-based payments cover a smaller part of the total income. The different levels of health care are reimbursed by different systems, where primary health care earnings are mainly dependent on registered patients, number of patient visits, and the diagnoses of each patient (Analytics, 2020).

Public health care in Sweden is decentralized in 21 regions, where Region Västra Götaland is the county council responsible for providing public health care in the territory Västra Götaland in Sweden. Västra Götaland inhabits a sixth of the Swedish population – 1.7 million people. The region is both rural and urban and contains Sweden’s second largest city, Gothenburg, with an inner-city population of around 580 000 in 2019. Since 2014, all publicly funded health care in Region Västra Götaland is reported to a government-controlled and supervised health care register (VEGA) (Regionfakta, 2019; Analytics, 2020).

The prevalence of diagnosed specific back pain among patients accessing health care is still not known in Sweden due to lack of data within the primary health care sector. These data are now available through the VEGA register. The knowledge would be of great significance to treat, handle, and mitigate health and economic implications caused by back pain. The aim of this study was therefore to investigate the annual prevalence of diagnosed specific back pain (Appendix 1) in Region Västra Götaland, Sweden, between 2014 and 2019, and to examine differences between health care levels, sex, age, and M48-, M50-, and M51 sub-classifications.

## Materials and methods

Data were extracted from the VEGA register between 2014 and 2019. The aggregated data covered all patients of all ages in all public-funded health care establishments in Västra Götaland diagnosed with specific back pain (Appendix 1). Other variables withdrawn from the register were sex, age, sub-classification (of M48, M50, and M51), the health care level in which the ICD-10 code was diagnosed, and number of health care visits on every level (primary health care, rehabilitation care, specialized outpatient care, and inpatient health care). The one-year prevalence was defined as the number of individuals given the actual ICD-10 code in proportion to the population in Region Västra Götaland that year. The same patient may receive a diagnosis from more than one health care level (primary, rehabilitation, specialized inpatient, and outpatient care) throughout the year. In the VEGA register, however, the health care levels are separated from each other. Even within every health care level, looking at the number of individuals, the register adjusts so that the same patient is only accounted for once annually even though the patient received more than one of the M48, M50, and M51 codes. Microsoft Excel and the Chi-square test were used for the statistical analyses. Ethical approval has been applied for and accepted by the Swedish Ethical Review Authority: EPM DNR 2020-03722.

## Results

**Figure 1.**
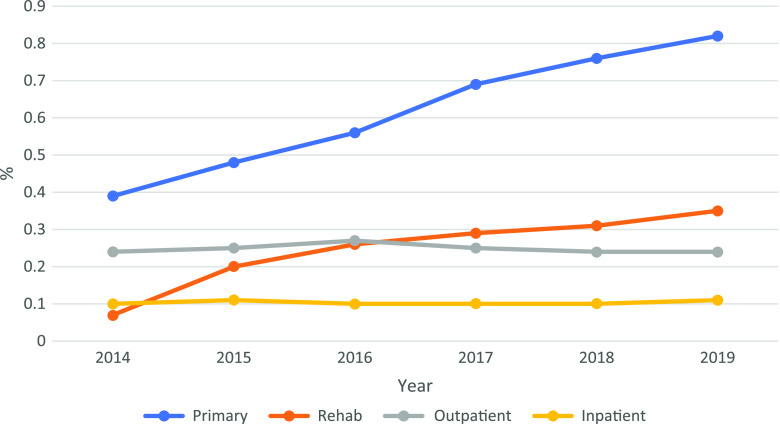
The one-year prevalence of all diagnosed specific back pain diagnoses stratified by year and health care level.

The one-year prevalence for all diagnosed specific back pain in both PHC and rehabilitation care has constantly increased from 2014. As demonstrated in Figure 1, the diagnosed prevalence within PHC in 2019 was 0.82%, which is an increase of 110% from 2014 (*P* < 0.01, CI 104%–116%). For rehabilitation care, the annual diagnosed prevalence in 2019 was 0.35%, resulting in a 418% increase from 2014 (*P* < 0.01, CI 386%–452%). In specialized health care, the diagnosed prevalence during 2014–2019 has remained relatively unchanged.

The majority (87%) of all health care visits due to diagnosed specific back pain were attended in primary health care. Most of the health care visits in 2019 were conducted within rehabilitation care, where every patient with specific back pain diagnose visits a physiotherapist 6.85 times on average. Women conduct more visits than men in all health care levels, which is demonstrated in Table 1.

**Table 1.** The number of health care visits per patient with diagnosed specific back pain stratified by health care level in 2019

	PHC			Rehab			Outpatient			Inpatient		
	Female	Male	Total	Female	Male	Total	Female	Male	Total	Female	Male	Total
**Total visits**	17 834	13 654	31 488	25 487	16 437	41 924	4319	3749	8068	1436	1214	2650
**Total patients**	8012	6131	14 143	3559	2558	6117	2129	1953	4082	1111	865	1976
**Mean visit/patient**	2.23	2.23	2.23	7.16	6.43	6.85	2.03	1.92	1.98	1.29	1.40	1.34

Of all patients with diagnosed specific back pain within PHC and rehabilitation care, 64% were diagnosed with ICD-10 codes M48.0 (Spinal stenosis) or M51.1K (Lumbar disc herniation with radiculopathy), making them the most common sub-classifications of all diagnoses (Appendix 1) for both sexes. In total, 70.2% of the patients were diagnosed with their M48.0 code within primary health care, whereas for M51.1K, it was 64.7%.

**Figure 2.**
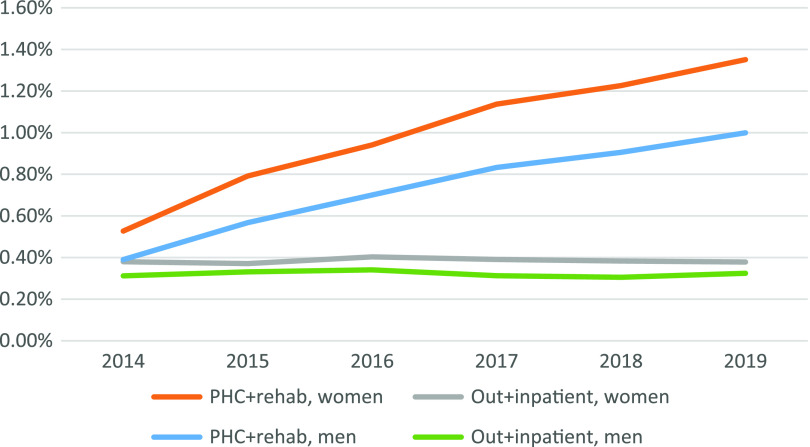
Diagnosed one-year prevalence of all diagnosed specific back pain diagnoses stratified by sex and health care level in 2019.

As demonstrated in Figure 2, the annual prevalence of total diagnosed specific back pain among both women and men increased each year from 2014 to 2019, as did the difference between the sexes. The one-year prevalence is higher among women in all the studied years, being more pronounced in primary health care than in specialized health care. In 2019, the one-year prevalence of all diagnosed specific back pain in primary health care was 1.35% among women and 1% among men, a difference of 35% (*P* < 0.01, CI 32%–39%). The one-year prevalence of M51.1K and M48.0 within PHC and rehabilitation care was statistically significantly higher among women in all the studied years, being more pronounced for the latter.

**Figure 3.**
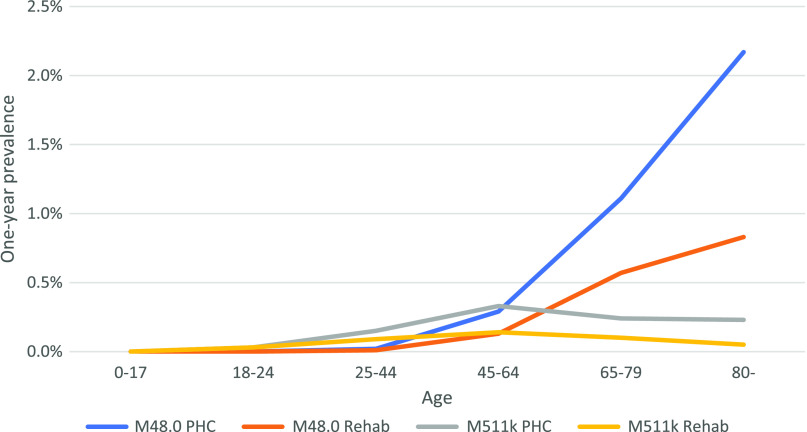
Diagnosed one-year prevalence of M48.0 and M51.1K in 2019 in primary health care. Stratified by age and PHC or rehabilitation centres.

Figure 3 demonstrates the age distribution of M48.0 and M51.1K in 2019 in primary health care. The diagnosed one-year prevalence of M48.0 increases with age at all health care levels. In primary health care and rehabilitation care, M51.1K is more common in younger patients, having the highest figures in the 45–64 years category. In specialized health care in 2019, M51.1K was most prominent in not only the 45–64 years category but also at 25–44 years of age.

## Discussion

In Region Västra Götaland 2019, the one-year prevalence of diagnosed specific back pain in primary health care was 1.17% (PHC 0.82% and rehabilitation care 0.35%). This is an increase by 156% (0.46% to 1.17%) from 2014 to 2019. Interestingly, the annual diagnosed prevalence is more than twice as much within PHC compared to rehabilitation care. Physiotherapy and patient education are in clinical guidelines recommended to be first-line treatments for back pain including specific benign spinal pathologies. Improving direct access to health care practitioners such as physiotherapists is therefore of importance (Schroder *et al.*, 2021).

The most frequently occurring sub-classifications at all health care levels for both sexes were M48.0 (spinal stenosis) and M51.1K (lumbar disc herniation with radiculopathy). Previous studies have shown higher prevalence (Schroeder, Kurd & Vaccaro, 2016; Arts *et al.*, 2019; Jensen *et al.*, 2020), which may be explained by study design, this study being register-based with a large sample size, and others being studies of smaller size using a broad range of definitions and measures of prevalence, which might not make them comparable. Furthermore, the large range in reported prevalence is also dependent on diagnostic criteria, both clinically and radiologically, for which there is high variety (Jensen *et al.*, 2020). Symptoms of back pain might also be undiagnosed or misdiagnosed. For example, symptoms of spinal stenosis are frequently dismissed as part of the ageing process and rather diagnosed with other ICD-10 codes covering back pain, such as M54.5 (lumbago). This pattern is also likely for similar degenerative joint conditions such as osteoarthritis (Turkiewicz *et al.*, 2015).

The annual increase in diagnosed one-year prevalence from 2014 to 2019 may be explained by several factors, such as the inclusion of rehabilitation care (primarily physiotherapy) in the VEGA register 2014. Further, a recent report on the registration of diagnoses in Region Västra Götaland shows a clear general increase for the total number of diagnoses registered in primary health care since 2010 and in particular since 2014/2015, which corresponds to our results. This indicates a change in behaviour among medical staff, with a probable increased awareness of the economic reimbursement linked to different diagnoses (Analytics, 2020; Sjöström & Johansson, 2020). A change in the sociodemographic setting in Region Västra Götaland since 2015, with the increase of immigration from Syria, Afghanistan, and India (Sternvik, 2019), could also be another confounding factor. According to a governmental analysis, immigrants, despite adjustments for education and income, were 2.6 times more likely to suffer from back pain compared to the general population (Anna Kjellström, Maria Telemo & Henry 2019; Analytics, 2020).

In accordance with previous studies (Melancia, Francisco & Antunes, 2014; Schroeder, Kurd & Vaccaro, 2016), the diagnosed one-year prevalence of M48.0 increases with age in primary health care, rehabilitation care, and specialized care. Spinal stenosis usually develops over time, degenerative changes being the main cause of the narrowing of the spinal canal. The diagnosed one-year prevalence of M51.1K (lumbar disc herniation with radiculopathy) peaks earlier, in the 45–64 years group, as concluded in a previous review (Jordan, Konstantinou & O’Dowd, 2011).

The current study found that diagnosed specific back pain is 33% more common among women than in men (0.94% vs 0.71% in 2019) in PHC, and in rehabilitation care, it was 41% (0.42% vs 0.29%). The sex difference is more pronounced for M48.0 (spinal stenosis) than for M51.1K (lumbar disc herniation with radiculopathy). Sex as a risk factor for disc herniation has been debated with various conclusions. Some claim men are overrepresented, whilst others state that the male sex is not a predictor (Jordan, Konstantinou & O’Dowd, 2011; Huang *et al.*, 2016). Studies have however shown that women have significantly worse baseline symptoms before surgery, which indicate that women undergoing discectomy potentially are referred and evaluated by different standards compared to men. It is also possible that the burden of symptoms between men and women is experienced differently, even though the degree of pain and disability are the same for the two sexes (Weinstein *et al.*, 2008; Pearson, 2016). Generally, there is a higher prevalence among women to seek public medical aid that have been documented both in general (Bo Palaszewski, 2019) and for back pain (Thoreson, Aminoff & Parai, 2020). A systematic review has though concluded that female sex was only a minor cause of care seeking due to back pain, compared to the most prominent cause that was the level of disability that the patient endured (Ferreira *et al.*, 2010). This emphasises the importance of a patient-focused care, and perhaps a more frequent use of pain charts or similar medical tools to increase the probability of an unbiased assessment (Bertilson *et al.*, 2007; Bertilson *et al.*, 2010).

Interestingly, the diagnosed one-year prevalence in specialized health care has remained constant during all the studied years. Furthermore, looking at the decompressive surgeries achieved in Region Västra Götaland during 2019, women are operated on to the same extent as men (Ludvigsson *et al.*, 2011; Socialstyrelsen, 2020). Since the annual prevalence of diagnosed specific back pain was considerably higher among women in primary health care, this raises important questions. Health care is perhaps not equal when it comes to sex, or else, men and women are perchance treated differently. Are women referred to surgeons less readily or are less enthusiastic about surgery?

## Strengths and limitations

A major strength of this study is the reliability of the data in the VEGA register. The register is government-controlled, and it is mandatory to register; thus, the public health care coverage is 100%. Furthermore, the sample size (frame population) was large. There may of course be visits that do not have an ICD-code registered, which would affect the prevalence. However, since primary health care earnings are dependent on diagnoses (ICD-codes) of each patient and the results include all unique patients that have been diagnosed with the corresponding ICD-code at least once in a year, this is probably not a common problem.

In this study, specific back pain is referred to as spinal stenosis and disc herniation. Other diagnoses, such as tumours in the spine, spondylolisthesis, cauda equina syndrome, ankylosing spondylitis, and other inflammatory diseases, are not covered in this study. The risk of being undiagnosed or misdiagnosed must also be taken into consideration. Back pain symptoms can be classified elsewhere within the ICD system, for instance as nonspecific back pain (M54.9) or lumbago (M54.5), which are not included in this study.

## Clinical implications

Important clinical findings of this study were an increasing one-year prevalence of diagnosed specific back pain in primary health care (PHC and rehabilitation care). Fifty-seven percentage of the patients that visited PHC did not receive physiotherapy treatments. These data suggest the need for implementing a standardized clinical pathway for back pain in Swedish health care, to help patients access the right health care at the right time. Women were overrepresented in all the studied years but received surgically interventions to the same extent as men, even though women’s baseline symptoms have been reported being significantly worse. This highlights the importance of a structured and accurate patient history and examination, with certain emphasis towards women, not to deprive them of treatment options.

## Conclusion

The one-year prevalence for all diagnosed specific back pain has constantly increased from 2014, resulting in an annual diagnosed prevalence of 0.82% within primary health care centres and 0.35% for rehabilitation care in 2019. The difference indicates more patients need to see a physiotherapist to receive important treatments. Diagnosed specific back pain is more common among women than men, which interestingly is not reflected in reported surgical interventions. This underlines the importance of a structured and accurate patient history and examination, with certain emphasis towards women, not to deprive them of treatment options.
